# Regular exercise counteracts circadian shifts in core body temperature during long-duration bed rest

**DOI:** 10.1038/s41526-020-00129-1

**Published:** 2021-01-05

**Authors:** Stefan Mendt, Hanns-Christian Gunga, Dieter Felsenberg, Daniel L. Belavy, Mathias Steinach, Alexander C. Stahn

**Affiliations:** 1 Charité – Universitätsmedizin Berlin, corporate member of Freie Universität Berlin, Humboldt-Universität zu Berlin, and Berlin Institute of Health, Institute of Physiology, Center for Space Medicine and Extreme Environments Berlin, Berlin, Germany; 2 Charité – Universitätsmedizin Berlin, corporate member of Freie Universität Berlin, Humboldt-Universität zu Berlin, and Berlin Institute of Health, Centre for Muscle and Bone Research, Berlin, Germany; 3 Institute for Physical Activity and Nutrition, School of Exercise and Nutrition Sciences, Deakin University, Geelong, Australia; 4 Department of Psychiatry, Perelman School of Medicine, University of Pennsylvania, Philadelphia, PA USA

**Keywords:** Neuroscience, Physiology

## Abstract

With NASA’s plans for the human exploration of Mars, astronauts will be exposed to mission durations much longer than current spaceflight missions on the International Space Station. These mission durations will increase the risk for circadian misalignment. Exercise has gained increasing interest as a non-pharmacological aid to entrain the circadian system. To assess the potential of exercise as a countermeasure to mitigate the risk for circadian disorders during spaceflight, we investigated the effects of long-term head-down tilt bed rest (HDBR) with and without exercise on the circadian rhythm of core body temperature. Core body temperature was recorded for 24 h using a rectal probe in sixteen healthy men (age: 30.5 ± 7.5 years (mean ± SD)) after 7 days and 49 days of HDBR. Five participants underwent HDBR only (CTR), five participants underwent HDBR and performed resistive exercises (RE), and six participants underwent HDBR and performed resistive exercises superimposed with vibrations (RVE). The exercise was scheduled three times per week. CTR showed a phase delay of 0.69 h. In contrast, both exercise groups were characterized by a phase advance (0.45 h for RE and 0.45 h for RVE; *p* = 0.026 for interaction between time and group). These findings suggest that resistive exercise (with or without vibration) may also serve as a countermeasure during spaceflight to mitigate circadian misalignments. The results could also be important for increasing awareness about the role of circadian disorders in long-term bedridden patients.

## Introduction

Shift work, traveling through several time zones in a relatively short time, and spaceflight can challenge the temporal adaptation of the circadian rhythm to a 24-h day. Failure to properly entrain the body’s internal clock leads to circadian misalignment, which can impair mental^1,2^ and physical^3,4^ performance, and have considerable adverse long-term effects on health and well-being^5–9^. Astronauts are particularly vulnerable to circadian disorders because of shift work, high workloads, increased stress levels, irregular day and night cycles, and altered physical activity levels^10^. Recent data suggest that circadian misalignment occurs on about 20% of days during standard missions on the International Space Station, resulting in about 1 h loss in sleep per night^11^. Circadian disruptions and sleep loss could have significant consequences on the health, safety, operational performance, and success of astronaut crews, stressing the need to assess effective countermeasures to mitigate circadian disorders during spaceflight^12^. This will be even more critical for long-duration exploratory missions, including missions to Mars because the prolonged confinement and isolation may further challenge regular behavioral routines (i.e., sleep/wake and rest/activity cycles) and thus potentially exacerbated circadian disruption^13^.

During these missions, astronauts need to engage in daily exercise routines to minimize the effects of cardiovascular deconditioning and muscle and bone loss associated with weightlessness^14,15^. An increasing number of studies suggest that in addition to light^16^ and social routines such as regular meal times^17^, physical exercise^18^ could be a potent “zeitgeber” for entraining the circadian system. The role of physical exercise to mitigate circadian misalignment during spaceflight is currently not well understood.

Head-down tilt bed rest (HDBR) is considered an excellent terrestrial model to mimic some of the stressors associated with spaceflight on the human body^19,20^. Whereas the influence of gravity is still present, 6 degrees HDBR causes physiological responses similar to those naturally occurring in space, including, but not limited to a fluid shift to the upper body, the absence of changes in posture and working against the force of gravity, and limited locomotion and reduced physical activity. Given the highly controlled and standardized settings of HDBR, HDBR has been used as a model to assess the efficacy of countermeasures against muscle and bone loss, and cardiovascular deconditioning^19^. Previous studies suggest that HDBR also induces a phase delay of circadian rhythms (range of average phase delays: ~1.3 to 4.0 min/day)^21–23^. However, it is unknown whether regular exercise could mitigate such effects.

To address this gap, we investigated the effects of 60 days of 6 degrees HDBR with and without regular resistive exercise on the circadian rhythm of core body temperature. The study was conducted as part of the Berlin Bed Rest Study 2 (BBR2-2)^24^. Based on a previous experiment investigating the effects of time of exercise on a circadian rhythm during 3-day horizontal bed rest^25^, we predicted that HDBR without exercise would induce a phase delay of circadian rhythm, which would be counteracted by the exercise program.

## Results

### Data quality and demographics

To assess the effect of HDBR with and without resistance exercise as a countermeasure on the circadian timing system, rectal core body temperature (CBT) was continuously recorded for 24 h after one week (HDBR7) and after 7 weeks (HDBR49) of HDBR. Data are reported for a total of *N* = 16 healthy, young men, who were randomly allocated to one of the following subgroups during the baseline data collection of BBR2-2: HDBR only (CTR) (*n* = 5), HDBR plus resistive exercise (RE) (*n* = 5) and HDBR plus resistive exercise combined with vibrations (RVE) (*n* = 6). Detailed demographics for each group are provided in Table 1.

**Table 1:** Participant characteristics at baseline.

Group	*n*	Age (years)	Weight (kg)	Height (cm)
CTR	5	28.2 ± 5.8	79.3 ± 5.3*	176.9 ± 3.1
RE	5	31.5 ± 6.3	69.6 ± 4.2	176.8 ± 3.1
RVE	6	31.6 ± 10.1	80.4 ± 7.0*	179.2 ± 5.0

Values are means and standard deviations.

*Significantly different from RE (*p* < 0.05, Wilcoxon’s signed-rank test).

**Fig. 1:**
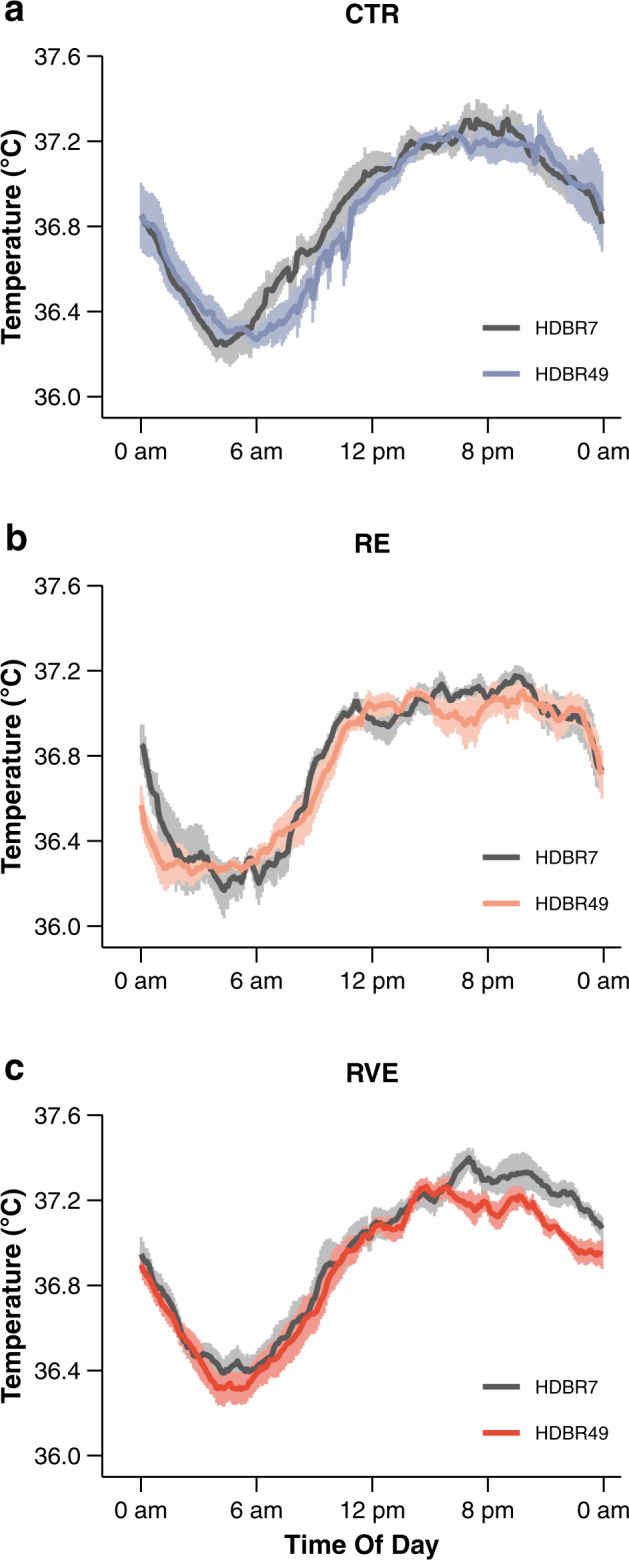
Average 24-h rectal temperature profiles on day 7 (dark solid line) and day 49 (colored solid line) of strict head-down tilt bed rest (HDBR). **a** CTR performed HDBR only (*n* = 5), **b** RE performed HDBR plus three times per week high-load resistive exercise (*n* = 5), **c** RVE performed the same exercise as RE, but with additional superimposed whole-body vibrations (*n* = 6). Vertical bars denote standard errors.

All temperature profiles showed a clear daily rhythm, i.e., the lowest temperature in the early morning and the highest in the early evening, and a good degree of parallelism with a flattening of the temperature profile around the afternoon to evening (Fig. 1).

### Changes in diurnal rhythms of rectal temperature

Visual inspection revealed a slight phase shift in CTR on HDBR49 (delayed increase in CBT in the morning). Individual data temperature profiles were approximated by a cosinor model to determine mesor, amplitude, and acrophase. All model fits confirmed the existence of a rhythm in the time series (*p* < 0.0001). Descriptive means and standard errors for mesor, amplitude, and acrophase at HDBR7 and HDBR49 for each group are provided in Table 2. Data at HDBR7 were comparable to cosinor rhythm parameters of CBT observed in healthy young participants^26^.

**Table 2:** Effect of head-down tilt bed rest (HDBR) on 24-h rectal temperature rhythm.

Parameter	Group	HDBR7	HDBR49
Mesor (°C)	CTR	36.86 ± 0.05	36.83 ± 0.06
	RE	36.78 ± 0.03	36.75 ± 0.02
	RVE	36.95 ± 0.05	36.87 ± 0.03
Amplitude (°C)	CTR	0.48 ± 0.04	0.49 ± 0.03
	RE	0.44 ± 0.07	0.46 ± 0.04
	RVE	0.46 ± 0.04	0.44 ± 0.04
Acrophase (h)	CTR	16.95 ± 0.53	17.65 ± 0.49
	RE	17.05 ± 0.32	16.60 ± 0.55
	RVE	17.52 ± 0.26	17.07 ± 0.43

Data are means and standard errors. Circadian parameters were determined by cosinor analysis using 24-h core body temperature recordings. Data were collected on day 7 (HDBR7) and day 49 (HDBR49) of HDBR. CTR performed HDBR only (*n* = 5). RE performed HDBR plus three times per week high-load resistive exercise (*n* = 5). RVE performed HDBR and the same exercise as the RE group, but with additional superimposed whole-body vibrations (*n* = 6).

**Fig. 2:**
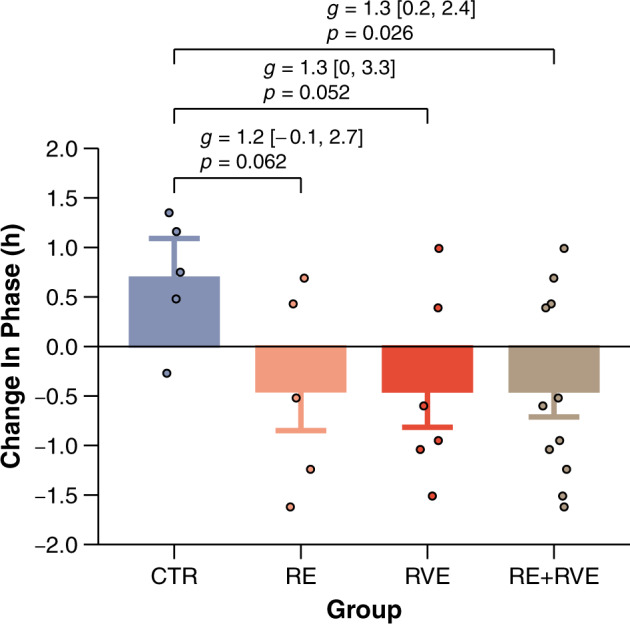
Changes in the circadian phase of rectal temperature from day 7 to day 49 of head-down tilt bed rest (HDBR). CTR, control group, i.e., bed rest only HDBR only (*n* = 5). RE, bed rest combined with resistance exercise (*n* = 5). RVE, bed rest combined with resistive exercise superimposed with whole-body vibrations (*n* = 6). Data are presented as estimated means and standard errors. Individual data are shown as dots. Positive numbers are defined as phase delays and negative numbers as phase advances. Effect sizes are Hedge’s *g* with 95% confidence intervals.

Mesor and amplitude remained nearly unchanged between HDBR7 and HDBR49 across all groups (Table 2). This was confirmed by mixed linear models that did not reveal any significant main effects for time and group or their interaction (Table 3). Mean acrophase ranged between 16.60 and 17.65 h for all groups (Table 2). The non-exercise group (CTR) showed opposite changes in acrophase from HDBR7 to HDBR49 compared to both exercise groups (RE and RVE). The CTR group was characterized by a phase delay, whereas we observed a phase advance in RE and RVE. These changes were quantified by a significant interaction between time and group for acrophase (*F*_1, 14_ = 6.19, *p* = 0.026). RE and RVE were characterized by a comparable phase advance (estimated mean ± SE: −0.45 ± 0.40 h for RE and −0.45 ± 0.35 h for RVE), and their interaction with CTR showed a strong effect for both subgroups (Hedge’s *g* > 1.2), almost reaching the level of significance (RE vs. CTR: −1.15 ± 0.56 h, *t*(13) = 2.04, *p* = 0.062; RVE vs. CTR: −1.15 ± 0.54 h, *t*(13) = 2.14, *p* = 0.052) (Fig. 2).

**Table 3:** Effects for linear mixed models examining the effects of time and group on mesor, amplitude, and acrophase.

Parameter	Factor			*df*_1_	*df*_2_	*F*	*p*
Mesor		Time	1		14	3.23	0.094
		Group	1		14	<0.01	0.969
		Time × Group	1		14	0.17	0.683
Amplitude		Time	1		14	0.01	0.908
		Group	1		14	0.52	0.484
		Time × Group	1		14	0.10	0.759
Acrophase		Time	1		14	0.27	0.609
		Group	1		14	0.20	0.658
		Time × Group	1		14	6.19	0.026

Results are based on 2 × 2 mixed-model ANOVA. Time and group were modeled as fixed factors. Time was characterized by the levels HDBR7 and HDBR49. Group was entered with two levels, comparing CTR (*n* = 5) to bed rest plus resistive exercise irrespective of the exercise protocol (pooled data from RE and RVE, *n* = 11).

*df*_*1*_, numerator degrees of freedom; *df*_*2*_, denominator degrees of freedom; *F,* *F*-statistic; *p,* *p*-value.

## Discussion

We here investigated the effects of long-duration head-down tilt bed rest (HDBR) with and without resistance exercise on the circadian rhythm of core body temperature. We found that HDBR induces a phase delay of circadian rhythm after 49 days of HDBR. This phase shift was counteracted by high-intensity resistance exercise training. Whether the resistance training was superimposed by whole-body vibrations or did not mediate this effect.

The suprachiasmatic nucleus (SCN) in men has an intrinsic period of 24 h and 11 min^27^. Using external temporal cues, so-called “zeitgeber”, the SCN entrains the circadian rhythm to 24-h day-night cycles^28^. Environmental conditions during BBR2-2 were similar to those encountered in normal everyday life including conventional lighting (i.e., participants were not exposed to special lightning conditions; room light could be adjusted according to personal preferences except during night), regular sleep-wake cycles, regular meal times, limited social contacts (i.e., restricted to staff members and other participants), and no temporal isolation (e.g., participants had access to clocks and were exposed to natural daylight through a window). These daily photic and non-photic stimuli are well-known to entrain the SCN to the 24-h day-night cycle^29^.

Yet, we still observed a phase delay of 42 min after 42 days of HDBR. This observation is in line with previous bed-rest studies. During a 7-day HDBR study acrophase of body temperature rhythm was delayed by 18 min^21^. Likewise, at the end of a 17-day HDBR study the circadian phase of rectal temperature was delayed by ~40 min^23^. This is also supported by a study investigating the effect of HDBR on melatonin. After 11 days of HDBR the average melatonin secretion peak was delayed by 16 min^22^. The average phase delay per day reported in these studies and our findings are highly comparable (range: 1–4 min/day) despite considerable differences between studies relative to lighting conditions, outcome measures, study durations, and data collection schedules. It should be noted though that the phase shift was not statistically different from zero in these studies, which we attribute to lack of power due to considerable inter-individual variation between subjects, relatively small mean phase delays per day, and small sample sizes.

Immobilization is characterized by inactivity and the absence of changes in posture during the day. Postural changes, physical activity levels, and diurnal variations in sleep-wake cycles are critical for entrainmening the circadian clock to 24 h. Studies using small rodents substantiate our assumption. Restriction of locomotor activity dampens the entrainment of the circadian clock ^30,31^. Bed rest participants also hardly change their body position from rest-activity to sleep and vice versa. Phase delays have been also observed in horizontal bed rest studies^19,25^, suggesting that immobilization rather than head-down tilt posture account for the phase shifts in circadian rhythm. In RE and RVE the circadian phase was advanced, i.e., shifted to an earlier time of day by about 0.5 h. Considering the HDBR-induced phase delay observed in the CTR group (about 0.7 h) this corresponds to a phase advance of about 1.2 h in the exercise groups. This effect is likely to be explained by the exercise program since the phase-shifting effect was similar in both exercise groups and the remaining conditions were similar across participants during HDBR. Collectively, these data demonstrate that resistance exercise performed three times per week between 10 am and 1 pm induces a phase advance.

Our findings are supported by various studies investigating the acute effects of exercise on the circadian system. Buxton and colleagues examined the effect of a single exercise session (1 h of high-intensity exercise on a stair climber) at morning, afternoon, evening, and night on the melatonin rhythm in a 3-day horizontal bed rest study with participants in recumbent posture^25^. Their data suggested a switch from a phase delay to a phase advance for exercise sessions performed 12 h after melatonin secretion onset^25^. These data would predict exercise-induced phase advances for exercise scheduled after 11 am. Similar conclusions are supported by Edwards et al., who investigated the effects of a single bout of 30-min cycle ergometer exercise on rectal temperature^32^. They reported phase advances when exercise was performed three to eight hours after CBT reached its minimum (CBT_min_)^32^. With a CBT_min_ around 5 am^33^ this would correspond to a phase advance associated with exercise between 8 am and 1 pm. These data are also supported by Miyazaki and colleagues who studied the timing of daily aerobic exercise on phase shifts exercise in participants who followed a 23 h 40 min sleep-wake cycle for 15 days^34^. A phase advance was observed when exercise was performed 6–9 h after the peak of plasma melatonin^35^. Given that plasma melatonin peak coincides with CBT_min_ around 5 am^33^, these data would predict a phase advance of CBT when exercise was performed between 11 am and 2 pm. Youngstedt and coworkers investigated the effects of 1 h of moderate treadmill exercise on three consecutive days at eight different day and night times in over 100 participants who followed a 90-min laboratory ultra-short sleep-wake cycle^36^. The authors reported a phase advance of 6-sulphatoxymelatonin when exercise was carried out between 7 am and 4 pm, and phase delays when exercising between 7 pm and 4 am^36^. These findings are in line with our data showing a phase advance of core body temperature in response to regular exercise performed between 10 am and 1 pm.

Our findings may be attributed to the effects of resistive exercise on the molecular clocks in the periphery^37,38^. One-legged resistive exercise, for example, alters the gene expression of clock genes in skeletal muscle compared to the non-exercised leg^39^. Whether such changes are then also reflected in the circadian central pacemaker (i.e., SCN), is currently unclear^40^. The roles and interactions of exercise intensity, type, and duration on the circadian timing system are also not well understood. This is exacerbated by the fact that it is unclear to what extent exercise alone is responsible for a phase shift. The above-mentioned studies were performed under light conditions which are known to affect the circadian system (exceeding 15 lux)^41^. Exercise activates the sympathetic nervous system causing pupils to dilate. In turn, larger amounts of light can be absorbed by the retina, which could contribute to the phase advance observed in the exercise groups^42,43^.

Although we observed opposite phase-shift effects in a highly standardized HDBR study (i.e., BBR2-2), our experiment is not without limitation. A higher data sampling could substantiate whether circadian shifts persist with the progress of bed rest. We have also not collected any behavioral data that could reflect the changes in circadian rhythms. Given the phase-shift effects in the present and previous HDBR studies we would expect these effects to be rather small though. Mental performance, for example, correlates with the circadian rhythm of core body temperature^44,45^. In HDBR studies of 60 and 90 days, no negative effects on cognitive functioning were found^46^. However, a general statement about the influence of bed rest on mental performance is hampered by differences in the administrated cognitive test, practice effects, task difficulty, and participants’ characteristics^45,47^. Lipnicki and Gunga^47^ reviewed 17 bed rest studies varying in duration between 7 and 70 days. They concluded that reported results vary considerably between studies, ranging from a detrimental effect to improvements in cognitive functions. Moreover, exercising during HDBR has been shown to mitigate some of the negative effects of HDBR on the brain^48^. It is possible that such effects correlate with circadian alterations in response to HDBR because the exercise and non-exercise groups experienced a phase shift in opposite directions. Furthermore, altered circadian rhythms have been observed in various mood disorders^49^. We have not collected any mood data, but previous HDBR studies reported adverse effects of bed rest on mental health. Ishizaki and colleagues observed increases in depressive and neurotic levels in a 20-day HDBR study. Notably, exercise training did not moderate this effect^50^. Likewise, Liu et al. demonstrated increases in depressive symptoms, and decreases in positive affect irrespective of physical exercise after 45 days of HDBR^51^. Participants of the present study were psychologically and medically healthy, and had no history of the psychological disease. A psychologist was available for consultation during the study^24^, and we are not aware of any reported adverse behavioral outcomes of the present study. Finally, we acknowledge that we did not acquire data on sleep duration and quality as part of the present experiment. In the 17-day HDBR study by Monk et al. participants showed poorer subjectively rated sleep quality and significantly longer nocturnal sleep onset latencies, but also some recovery with increasing duration of HDBR^23^. Future studies investigating the effects of bed rest on the circadian timing system should consider the relationship between phase shifts, sleep duration and quality, and neurobehavioral outcomes.

Taken together, our data suggest that long-duration bed rest weakens the entrainment of the circadian system, indicated by a phase delay of CBT. Regular exercise performed in the morning induces a phase advance and has the potential to counteract circadian misalignment. These findings underline the potential of exercise as a non-pharmacological aid to entrain the circadian system and help to raise awareness about the risk of circadian disorders in long-term bedridden patients. Resistive exercise may go beyond its role to support muscle and bone health, and also serve as a countermeasure to mitigate circadian misalignments associated with shift work, jet lag, and space travel. Future studies need to explore the underlying mechanisms and elucidate the interaction between light exposure and exercise on the circadian system.

## Methods

### Experimental design

The research was performed as part of the 2nd Berlin BedRest Study (BBR2-2) in a psychiatric ward on the medical campus Benjamin Franklin at Charité – Universitätsmedizin Berlin between 2007 and 2008. Details of the overall BBR2-2 study protocol are reported elsewhere^24^. Briefly, twenty-four healthy men (age: 32.5 ± 7.7 years (*M* ± *SD*)) were exposed to 60 days of 6 degrees head-down tilt (HDBR). They were randomly assigned via block randomization to one of the following groups: HDBR only (CTR), HDBR plus resistive exercise only (RE), and HDBR plus resistive exercise with superimposed whole-body vibration (RVE). To assess the effect of long-term exposure on the circadian system, 24-h rectal temperature data were collected on the 7th (HDBR7) and the 49th day of HDBR (HDBR49). To minimize the potential influence of bed rest associated with the beginning of bed rest (e.g., discomfort, pain, sleep difficulties)^52^ on the circadian rhythm, the first data recording was performed after 1 week of bed rest (HDBR7). Temperature measurements and exercise sessions were carried out on different days in order to avoid acute effects of exercise on the rectal temperature^53^. Weekly exercise sessions were conducted on Monday, Wednesday, and Friday, while data collections took place on Sunday. The study was approved by the local ethical committee of Charité – Universitätsmedizin Berlin.

### Participants

Twenty-four healthy men were enrolled in the study. Inclusion criteria comprised the absence of chronic diseases, sleep disorders, any kind of metabolic or hormonal disturbances, any metabolic diseases, and regular intake of medication. Additional details on exclusion and inclusion criteria are given elsewhere^24^. After the purpose, protocols, and known risks of the tests had been explained, all participants provided informed written consent before participating in the study. One participant of the RVE group felt unable to perform the exercises in HDBR and was assigned to the CTR group prior to the start of bed rest. Another participant from the RE group withdrew from the study after 30 days of bed rest for medical reasons^24^. All other twenty-three participants (CTR, *n* = 9; RE, *n* = 7; RVE, *n* = 7) completed 60 days of bed rest. Out of the fourteen participants from the two intervention groups, nine participants completed all 25 exercise sessions, three participants missed one session, one participant missed two sessions, and another participant missed four exercise sessions. Seven subjects did not meet the data quality criteria of core body temperature recordings and were excluded from the analyses (see section data analysis for details). The final data set comprised 16 participants with *n* = 5 in the CTR group, *n* = 5 in the RE group, and *n* = 6 in the RVE group. The anthropometric characteristics of the three groups are given in Table 1. There were no significant differences in age and height between groups (all *p* ≥ 0.60, Wilcoxon’s signed-rank test). The RE group, however, was characterized by significantly lower weight compared to CTR and RVE (Table 1).

### Experimental procedures

#### Bed rest protocol

Participants could change from supine to prone or lateral position as long as their legs were kept straight and the head remained lower than the feet. Activities were limited to a minimum level and performed in the head-down tilt position, including eating, urination, defecation, and personal hygiene. Diet was carefully controlled and served on a fixed schedule. Eating outside these times was not permitted. Participants were accommodated in pairs and in rooms with windows. A regular sleep/wake cycle was ensured by a normal light/dark cycle (i.e., natural daylight, domestic light, and by lights off between 11 pm and 7 am). Once the lights were turned out, participants were not allowed to perform any activity that interfered with sleeping. Compliance with the HDBR protocol was ensured by video monitoring.

#### Exercise protocol

Exercises were performed three times per week; on Monday, Wednesday, and Friday within one of four 45-min time-slots between 10 am and 1 pm. The training was scheduled such that participants rotated their time-slots from one exercise session to the next to avoid “time of day” effects on performance. Exercise sessions were structured as follows: (1) short warm-up (bilateral leg press with 50% of pre-bed rest maximum voluntary contraction); (2) bilateral leg press (75–80% of maximum); (3) single-leg heel raises (about 1.3 times of their HDBR1 body weight); (4) double-leg heel raises (about 1.8 times of their HDBR1 body weight); and finally (5) back and forefoot raise (performing hip and lumbar spine extension against gravity with ankle dorsiflexion; a force 1.5 times body weight was applied at the shoulders). The RVE group additionally received vibration with frequencies between 16 and 26 Hz, depending on the exercise. Each exercise session was performed on the Galileo Space exercise device (Novotec Medical GmbH, Pforzheim, Germany).

#### Core body temperature recordings

Rectal temperature was recorded using a flexible 4-mm NTC-thermal sensor (YSI 400 compatible, BlueTemp® products, bluepoint medical GmbH & Co, KG, Selmsdorf, Germany). The thermistor was inserted 5 cm past the anal sphincter. The temperature was continuously recorded using a mobile physiological monitoring system (HealthLab System, Koralewski Industrie Elektronik, Hambühren, Germany). The data was stored on the system and transferred to a personal computer after completion of the recording.

### Data analysis

Each raw temperature profile was visually inspected prior to further analyses. Changes in temperature related to hygiene activities or signal losses were manually excluded. Temperature profiles were then averaged over 6-min intervals. Data sets containing less than 85% of the 24-h recording time were discarded. Rhythm characteristics were estimated by fitting a sinusoidal function to the 24-h temperature profiles^54^. We applied a sinusoid with a single 24-h period in order to facilitate the comparison with previous bed rest studies^23^. Each time series was quantified by the fitted curve parameters mesor (mean of the fitted cosinor model), amplitude (half the difference between the highest and lowest value of the fitted curve), and acrophase (time point of the highest value of the fitted cosinor model). Data are reported as means and standard errors (SE) unless reported otherwise. Differences between HDBR7 and HDBR49 and intervention groups were assessed using mixed linear models with time (HDBR7 vs. HDBR49) and group (non-exercise vs. exercise) as fixed factors, and subjects as random factors with random intercepts for subjects. Normality and homogeneity were checked by visual inspection of plots of residuals against fitted values (*Q*–*Q* plots). Significant interactions were followed up by running another model to determine parameter estimates of the interactions between CTR and RE, and CTR and RVE, respectively. Covariance matrices were determined by restricted maximum likelihood (REML) estimation. *p*-values were obtained by using Satterthwaite’s approximation for denominator degrees of freedom. Effect sizes were reported as Hedge’s *g* and their 95% confidence intervals using bootstrapping^55^. The level of significance was set at 0.05 (two-sided) for all testing. All analyses and graphical illustrations were performed using the software package R^56^.

### Reporting summary

Further information on experimental design is available in the Nature Research Reporting Summary linked to this paper.

## Supplementary information

Reporting Summary Checklist

## Data Availability

The data that support the findings of this study are openly available in figshare at 10.6084/m9.figshare.12129534.

## References

[1] Basner M (2008). Effects of night work, sleep loss and time on task on simulated threat detection performance. Sleep.

[2] Chellappa SL, Morris CJ, Scheer FAJL (2019). Effects of circadian misalignment on cognition in chronic shift workers. Sci. Rep..

[3] Thun E, Bjorvatn B, Flo E, Harris A, Pallesen S (2015). Sleep, circadian rhythms, and athletic performance. Sleep. Med. Rev..

[4] Reilly T, Waterhouse J (2009). Sports performance: is there evidence that the body clock plays a role?. Eur. J. Appl. Physiol..

[5] Goel N, Basner M, Rao H, Dinges DF (2013). Circadian rhythms, sleep deprivation, and human performance. Prog. Mol. Biol. Transl. Sci..

[6] Perlis ML (2016). Nocturnal wakefulness as a previously unrecognized risk factor for suicide. J. Clin. Psychiatry.

[7] Scheer FA, Hilton MF, Mantzoros CS, Shea SA (2009). Adverse metabolic and cardiovascular consequences of circadian misalignment. Proc. Natl Acad. Sci. USA.

[8] Savvidis C, Koutsilieris M (2012). Circadian rhythm disruption in cancer biology. Mol. Med..

[9] Baron KG, Reid KJ (2014). Circadian misalignment and health. Int. Rev. Psychiatry.

[10] Guo J-H (2014). Keeping the right time in space: importance of circadian clock and sleep for physiology and performance of astronauts. Mil. Med. Res..

[11] Flynn-Evans EE, Barger LK, Kubey AA, Sullivan JP, Czeisler CA (2016). Circadian misalignment affects sleep and medication use before and during spaceflight. NPJ Microgravity.

[12] Mallis MM, DeRoshia CW (2005). Circadian rhythms, sleep, and performance in space. Aviat. Sp. Env. Med.

[13] Basner M (2013). Mars 520-d mission simulation reveals protracted crew hypokinesis and alterations of sleep duration and timing. Proc. Natl Acad. Sci. USA.

[14] Loehr JA (2015). Physical training for long-duration spaceflight. Aerosp. Med. Hum. Perform..

[15] Petersen N (2016). Exercise in space: the European Space Agency approach to in-flight exercise countermeasures for long-duration missions on ISS. Extrem. Physiol. Med..

[16] Husse J, Eichele G, Oster H (2015). Synchronization of the mammalian circadian timing system: Light can control peripheral clocks independently of the SCN clock: alternate routes of entrainment optimize the alignment of the body’s circadian clock network with external time. Bioessays.

[17] Patton DF, Mistlberger RE (2013). Circadian adaptations to meal timing: neuroendocrine mechanisms. Front. Neurosci..

[18] Lewis P, Korf HW, Kuffer L, Groß JV, Erren TC (2018). Exercise time cues (zeitgebers) for human circadian systems can foster health and improve performance: a systematic review. BMJ Open Sport Exerc. Med..

[19] Pavy-Le Traon A, Heer M, Narici MV, Rittweger J, Vernikos J (2007). From space to Earth: advances in human physiology from 20 years of bed rest studies (1986-2006). Eur. J. Appl. Physiol..

[20] Hargens AR, Vico L (2016). Long-duration bed rest as an analog to microgravity. J. Appl. Physiol..

[21] Samel A, Wegmann HM, Vejvoda M (1993). Response of the circadian system to 6 degrees head-down tilt bed rest. Aviat. Sp. Env. Med..

[22] Hurwitz S, Cohen RJ, Williams GH (2004). Diurnal variation of aldosterone and plasma renin activity: timing relation to melatonin and cortisol and consistency after prolonged bed rest. J. Appl. Physiol..

[23] Monk TH, Buysse DJ, Billy BD, Kennedy KS, Kupfer DJ (1997). The effects on human sleep and circadian rhythms of 17 days of continuous bedrest in the absence of daylight. Sleep.

[24] Belavý DL (2010). The 2nd Berlin BedRest Study: protocol and implementation. J. Musculoskelet. Neuronal. Interact..

[25] Buxton OM, Lee CW, L’Hermite-Baleriaux M, Turek FW, Van Cauter E (2003). Exercise elicits phase shifts and acute alterations of melatonin that vary with circadian phase. Am. J. Physiol. Regul. Integr. Comp. Physiol..

[26] Edwards B, Waterhouse J, Reilly T, Atkinson G (2002). A comparison of the suitabilities of rectal, gut, and insulated axilla temperatures for measurement of the circadian rhythm of core temperature in field studies. Chronobiol. Int..

[27] Duffy JF (2011). Sex difference in the near-24-hour intrinsic period of the human circadian timing system. Proc. Natl Acad. Sci. USA.

[28] Honma S (2018). The mammalian circadian system: a hierarchical multi-oscillator structure for generating circadian rhythm. J. Physiol. Sci..

[29] Mistlberger RE, Skene DJ (2005). Nonphotic entrainment in humans. J. Biol. Rhythm..

[30] Mrosovsky N, Reebs SG, Honrado GI, Salmon PA (1989). Behavioural entrainment of circadian rhythms. Experientia.

[31] Castillo C, Molyneux P, Carlson R, Harrington ME (2011). Restricted wheel access following a light cycle inversion slows re-entrainment without internal desynchrony as measured in Per2Luc mice. Neuroscience.

[32] Edwards B, Waterhouse J, Atkinson G, Reilly T (2002). Exercise does not necessarily influence the phase of the circadian rhythm in temperature in healthy humans. J. Sport. Sci..

[33] Rajaratnam SM, Arendt J (2001). Health in a 24-h society. Lancet.

[34] Miyazaki T, Hashimoto S, Masubuchi S, Honma S, Honma KI (2001). Phase-advance shifts of human circadian pacemaker are accelerated by daytime physical exercise. Am. J. Physiol. Regul. Integr. Comp. Physiol..

[35] Yamanaka Y (2006). Effects of physical exercise on human circadian rhythms. Sleep. Biol. Rhythms.

[36] Youngstedt SD, Elliott JA, Kripke DF (2019). Human circadian phase-response curves for exercise. J. Physiol..

[37] Aoyama S, Shibata S (2017). The role of circadian rhythms in muscular and osseous physiology and their regulation by nutrition and exercise. Front. Neurosci..

[38] Tahara Y, Aoyama S, Shibata S (2017). The mammalian circadian clock and its entrainment by stress and exercise. J. Physiol. Sci..

[39] Zambon AC (2003). Time- and exercise-dependent gene regulation in human skeletal muscle. Genome Biol..

[40] Yamanaka Y (2014). Differential regulation of circadian melatonin rhythm and sleep-wake cycle by bright lights and nonphotic time cues in humans. Am. J. Physiol. Regul. Integr. Comp. Physiol..

[41] Prayag, S. A., Münch, M., Aeschbach, D., Chellappa, L. S. & Gronfier, C. Light modulation of human clocks, wake, and sleep. *Clocks Sleep* **1**, 193–208 (2019). 10.3390/clockssleep1010017 PMC7185269 32342043

[42] Ishigaki H, Miyao M, Ishihara S (1991). Change of pupil size as a function of exercise. J. Hum. Ergol..

[43] Hayashi N, Someya N (2011). Muscle metaboreflex activation by static exercise dilates pupil in humans. Eur. J. Appl. Physiol..

[44] Valdez P, Reilly T, Waterhouse J (2008). Rhythms of mental performance. Mind Brain Educ..

[45] Blatter K, Cajochen C (2007). Circadian rhythms in cognitive performance: methodological constraints, protocols, theoretical underpinnings. Physiol. Behav..

[46] Seaton KA, Slack KJ, Sipes WA, Bowie KE (2009). Cognitive functioning in long-duration head-down bed rest. Aviat. Space Environ. Med..

[47] Lipnicki DM, Gunga H-C (2009). Physical inactivity and cognitive functioning: results from bed rest studies. Eur. J. Appl. Physiol..

[48] Friedl-Werner A, Brauns K, Gunga H-C, Kühn S, Stahn AC (2020). Exercise-induced changes in brain activity during memory encoding and retrieval after long-term bed rest. Neuroimage.

[49] Vadnie CA, McClung CA (2017). Circadian rhythm disturbances in mood disorders: insights into the role of the suprachiasmatic nucleus. Neural Plast..

[50] Ishizaki Y (2002). Changes in mood status and neurotic levels during a 20-day bed rest. Acta Astronaut..

[51] Liu Q, Zhou R, Chen S, Tan C (2012). Effects of head-down bed rest on the executive functions and emotional response. PLoS ONE.

[52] Meck JV, Dreyer SA, Warren LE (2009). Long-duration head-down bed rest: project overview, vital signs, and fluid balance. Aviat. Space Environ. Med..

[53] Lee SM, Williams WJ, Fortney Schneider SM (2000). Core temperature measurement during supine exercise: esophageal, rectal, and intestinal temperatures. Aviat. Sp. Env. Med..

[54] Refinetti R, Lissen GC, Halberg F (2007). Procedures for numerical analysis of circadian rhythms. Biol. Rhythm Res..

[55] Kirby KN, Gerlanc D (2013). BootES: an R package for bootstrap confidence intervals on effect sizes. Behav. Res. Methods.

[56] R Core Team. *R: A Language and Environment for Statistical Computing* (R Foundation for Statistical Computing, 2019).

