# Supplementary material for: Regular exercise counteracts circadian shifts in core body temperature during long-duration bed rest
Source: NPJ Microgravity. 2021 Jan 5;7:1. doi: 10.1038/s41526-020-00129-1 (PMC7785743; doi:10.1038/s41526-020-00129-1)
Supplement: Supplementary file 1 — Reporting Summary Checklist [file 41526_2020_129_MOESM1_ESM.pdf]

nature research

Corresponding author(s): Alexander C. Stahn

Last updated by author(s): Oct 7, 2020

Reporting Summary

Nature Research wishes to improve the reproducibility of the work that we publish. This form provides structure for consistency and transparency in reporting. For further information on Nature Research policies, see our Editorial Policies and the Editorial Policy Checklist.

Statistics

For all statistical analyses, confirm that the following items are present in the figure legend, table legend, main text, or Methods section.

n/a	Confirmed	
☐	☒	The exact sample size (n) for each experimental group/condition, given as a discrete number and unit of measurement
☐	☒	A statement on whether measurements were taken from distinct samples or whether the same sample was measured repeatedly
☐	☒	The statistical test(s) used AND whether they are one- or two-sided<br>*Only common tests should be described solely by name; describe more complex techniques in the Methods section.*
☒	☐	A description of all covariates tested
☐	☒	A description of any assumptions or corrections, such as tests of normality and adjustment for multiple comparisons
☐	☒	A full description of the statistical parameters including central tendency (e.g. means) or other basic estimates (e.g. regression coefficient) AND variation (e.g. standard deviation) or associated estimates of uncertainty (e.g. confidence intervals)
☐	☒	For null hypothesis testing, the test statistic (e.g. F, t, r) with confidence intervals, effect sizes, degrees of freedom and P value noted<br>*Give P values as exact values whenever suitable.*
☒	☐	For Bayesian analysis, information on the choice of priors and Markov chain Monte Carlo settings
☒	☐	For hierarchical and complex designs, identification of the appropriate level for tests and full reporting of outcomes
☐	☒	Estimates of effect sizes (e.g. Cohen's d, Pearson's r), indicating how they were calculated

Our web collection on statistics for biologists contains articles on many of the points above.

Software and code

Policy information about availability of computer code

Data collection	Core body temperatures were recorded using a mobile physiological monitoring system (HealthLab System, Koralewski Industrie Elektronik, Hambühren, Germany).
Data analysis	Data were analyzed with R (version 3.6.1) and the R packages BootES, lme4, lmerTest, and lsmeans.

For manuscripts utilizing custom algorithms or software that are central to the research but not yet described in published literature, software must be made available to editors and reviewers. We strongly encourage code deposition in a community repository (e.g. GitHub). See the Nature Research guidelines for submitting code & software for further information.

Data

Policy information about availability of data

All manuscripts must include a data availability statement. This statement should provide the following information, where applicable:

- Accession codes, unique identifiers, or web links for publicly available datasets
- A list of figures that have associated raw data
- A description of any restrictions on data availability

The data that support the findings of this study are openly available in figshare at http://doi.org/10.6084/m9.figshare.12129534.

Field-specific reporting

Please select the one below that is the best fit for your research. If you are not sure, read the appropriate sections before making your selection.

☒ Life sciences ☐ Behavioural & social sciences ☐ Ecological, evolutionary & environmental sciences

For a reference copy of the document with all sections, see nature.com/documents/nr-reporting-summary-flat.pdf

Life sciences study design

All studies must disclose on these points even when the disclosure is negative.

Sample size	Data were collected as part of the 2nd Berlin BedRest Study (BBR2-2). The primary objective of BBR2-2 was to evaluate different physical exercise programs to mitigate bone and muscle loss during 60 days of head-down tilt bed-rest. The organizers of BBR2-2 estimated the sample size based upon data from a previous bed rest study (Belavý, D. L. et al. The 2nd Berlin BedRest Study: protocol and implementation. J Musculoskelet Neuronal Interact 10, 207–219 (2010).
Data exclusions	Out of n=24 participants n=23 completed 60 days of head-down tilt bed rest. For the present study n=7 participants were excluded from data analyses because of incomplete data (data sets containing less than 85% of the 24-h recordings).
Replication	The study was not replicated.
Randomization	Participants were randomly assigned via block randomization into one of three groups 1) CTR: 60 days of head-down tilt bed rest, 2) RE: 60 days of head-down tilt bed rest plus regular resistive exercises, and 3) RVE: 60 days of head-down tilt bed rest plus regular resistive exercises superimposed by whole body vibration.
Blinding	Participants were not blinded to the intervention.

Reporting for specific materials, systems and methods

We require information from authors about some types of materials, experimental systems and methods used in many studies. Here, indicate whether each material, system or method listed is relevant to your study. If you are not sure if a list item applies to your research, read the appropriate section before selecting a response.

Materials & experimental systems

n/a	Involved in the study	
☒	☐	Antibodies
☒	☐	Eukaryotic cell lines
☒	☐	Palaeontology and archaeology
☒	☐	Animals and other organisms
☐	☒	Human research participants
☒	☐	Clinical data
☒	☐	Dual use research of concern

Methods

n/a	Involved in the study	
☒	☐	ChIP-seq
☒	☐	Flow cytometry
☒	☐	MRI-based neuroimaging

Human research participants

Policy information about studies involving human research participants

Population characteristics	Group characteristics of presented sub-samples of BBR2-2 were following:<br>CTR: n=5 men, 28.2 ± 5.8 years, 79.3 ± 5.3 kg, 176.9 ± 3.1 cm<br>RE: n=5 men, 31.5 ± 6.3 years, 69.6 ± 4.2 kg, 176.8 ± 3.1 cm<br>RVE: n=6 men, 31.6 ± 10.1 years, 80.4 ± 7.0 kg, 179.2 ± 5.0 cm<br>Note: Data are means and standard deviations
Recruitment	Participants were recruited via advertisements in local and regional newspapers, advertisements on public transport, posters placed at the hospital and universities. Out of N=1001 potential participants, N=24+1 were admitted to the study. Detailed information are provided in Belavý, D. L. et al. The 2nd Berlin BedRest Study: protocol and implementation. J Musculoskelet Neuronal Interact 10, 207–219 (2010)
Ethics oversight	The study was approved by the local ethical committee of Charité – Universitätsmedizin Berlin.

Note that full information on the approval of the study protocol must also be provided in the manuscript.
